# Case series of branch retinal vein occlusion secondary to rhegmatogenous retinal detachment and its surgical management

**DOI:** 10.1186/s12886-023-03244-1

**Published:** 2023-12-19

**Authors:** Youna Choi, Sung Jun Kim, Jae Jung Lee, Moosa Hasan Zaidi, Yong Un Shin, Ik Soo Byon, Ji Eun Lee, Sung Who Park

**Affiliations:** 1 Department of Ophthalmology, School of Medicine, Pusan National University, Pusan, South Korea; 2 Spencer Center for Vision Research, Byers Eye Institute, Stanford University, Palo Alto, CA USA; 3 Department of Ophthalmology, College of Medicine, Hanyang University, Seoul, South Korea; 4 Department of Ophthalmology, Pusan National University & Biomedical Research Institute, Pusan National University Hospital, 1-10 Ami-dong, Seo-gu, 602-739 Pusan, South Korea

**Keywords:** Branch retinal vein occlusion, Complication, Rhegmatogenous retinal detachment, Scleral buckling, Vitrectomy

## Abstract

**Background:**

To review cases of branch retinal vein occlusion (BRVO) secondary to rhegmatogenous retinal detachment (RRD) and its surgical management and presume their mechanism.

**Methods:**

Medical records of patients who underwent surgery for RRD between 2015 and 2019 at a single tertiary care center were retrospectively reviewed. New BRVO secondary to RRD or its surgical procedure was diagnosed based on the fundus examination and its clinical course.

**Results:**

A total of 734 RRD surgeries were performed for five years, and six cases of new BRVOs were noticed in the first year after surgery (incidence was 0.68%: six cases of BRVO / 734 cases of surgical RRD); five cases occurred after vitrectomy, and one occurred after scleral buckling. In three cases, retinal veins were presumed to already be partially occluded related due to a kink of the retinal vein seen before surgery. In the other three cases, the retinal veins were presumed to have incurred damage during vitrectomy.

**Conclusion:**

In the present cohort, RRD or its related procedures caused BRVO within a year of surgery at an incidence of 0.68%. The proposed mechanisms are kinks of the retinal vein on the detached retina and damage to the retinal vein during vitrectomy.

## Introduction

Known risk factors for branch retinal vein occlusion (BRVO) include advanced age [1], systemic vascular disorder [1, 2], abnormal blood viscosity, and hemostasis [2–5]. Known possible mechanisms of BRVO include mechanical compression of a branch vein by an artery at an arteriovenous crossing, degenerative changes in the vessel wall, and abnormal venous stasis [2]. Rhegmatogenous retinal detachment is a condition in which the neurosensory retina is separated from the retinal pigment epithelium in which retinal break and vitreous traction cause these. Vitrectomy or scleral buckling are main surgical procedures to treat RRD, and its incidence is reported 6.9 to 18.2 per 100,000 persons [6–9].

Our recent clinical experience has included cases of new BRVO occurring within months of rhegmatogenous retinal detachment (RRD) surgery. However, although previous BRVO is a well-known risk factor for RRD [10–12], no reports have shown that RRD surgery might be a risk factor for BRVO.

This study aimed to evaluate the relation of new BRVO to RRD surgery. Additionally, two possible mechanisms for increased risk of BRVO after RRD were explored.

## Material and methods

This case series complied with the tenets of the Declaration of Helsinki and was approved by the institutional review board of Pusan National University Hospital (approval no. H-2007–003-092).

The medical records of patients who underwent vitreoretinal surgery for RRD between March 2015 and December 2019 at Pusan National University, Busan, South Korea, were retrospectively reviewed.

Postoperative ultra-wide fundus photographs taken up to 12 months after surgery were reviewed.

A new BRVO was diagnosed based on fundus exam, showing multiple new intraretinal hemorrhages in the distribution of a large retinal vein and excluding other etiologies such as retinal vasculitis, drug toxicity, diabetic retinopathy. Fluorescein angiography (FA) was done to exclude other causes at clinician’s discretion. Eyes with a history of BRVO, uveitis, or moderate non-proliferative diabetic retinopathy or more severe were excluded.

The incidence of new BRVO after RRD surgery was analyzed. The demographics and clinical characteristics of patients who developed new BRVO and patients who did not develop new BRVO were reviewed, including age, sex, systemic disease, intraocular pressure, surgical methods, and preoperative best-corrected visual acuity (BCVA). The characteristics of each new BRVO were also reviewed, including its distribution, time to onset, and aggravation duration. The occlusive location was inferred based on the distribution of the flame-shaped hemorrhages. Preoperative fundus photographs were reviewed to identify any specific findings around the inferred occlusive location.

The time to onset was defined as the time interval from time of surgery to the time when the dot hemorrhages were first recognized. The duration of aggravation was defined as the duration from surgery to the hemorrhage peak.

### Surgical procedure

Surgical procedure was done by four surgeons. Scleral buckling was combined with cryoretinopexy in all cases and subretinal fluid was drained at each surgeon’s discretion. Twenty-five gauges pars plana vitrectomy was done using Constellation (Alcon Fort Worth, Tx, USA) under wide angle viewing system (Resight 700 Carl Zeiss Meditec AG, Jana, Germany). Room air tamponade was used in most cases and silicone oil was filled for the other cases according to our previous report [13]. Intraocular antibiotics was not used.

### Statistical analysis

The Mann–Whitney U test and Fisher’s exact test were used to compare the characteristics of patients who developed new BRVO after RRD surgery to patients who did not develop new BRVO after RRD surgery. Statistical significance was set at *P* < 0.05. All statistical analyses were performed using SPSS version 18.0 (SPSS Inc, IBM Company, Chicago, IL, USA).

## Results

The current study included 734 consecutive eyes treated for RRD, including 514 eyes treated with vitrectomy and 220 eyes treated with scleral buckling. New BRVO was observed in six eyes (0.68%): after vitrectomy in five eyes (incidence, 0.97%) and after scleral buckling in one eye (incidence, 0.46%). There was no significant difference among four surgeons regarding the incidence.

The clinical characteristics of the six patients are summarized in Table 1. The average age of the six patients who developed BRVO was 60.0 years (range, 48–74 years), which was greater than that of those who did not develop BRVO which was 53.6 years (range, 28–83 years), although this difference did not reach statistical significance (*p* = 0.093, Mann–Whitney U test). Three (50%) of the patients who developed new BRVO had hypertension or hyperlipidemia, compared to 176 (24%) of the patients who did not develop new BRVO (*p* = 0.1569, Fisher’s exact test). Among the six eyes that developed BRVO, the mean preoperative logMAR BCVA was 0.350 (Snellen 20/45, range 20/4000 ~ 20/25). In the preoperative examinations, posterior vitreous detachment was observed in four of the eyes (66.7%), and invasion of the retinal detachment into the fovea was observed in three of the eyes (50%). FA performed in four cases.

**Table 1:** Demographics of six eyes with branch retinal vein occlusion

	**Case 1**	**Case 2**	**Case 3**	**Case 4**	**Case 5**	**Case 6**
	**Mechanism I**			**Mechanism II**		
**Age, years**	48	63	74	63	64	48
**Gender**	Female	Female	Female	Female	Female	Male
**Hypertension or Dyslipidemia**	Hypertension	Dyslipidemia	Hypertension	None	None	None
**Affected side: Right or Left Eye**	Right	Right	Left	Left	Right	Left
**Preoperative visual acuity**	20/400	20/30	20/4000	20/25	20/30	20/200
**Final visual acuity**	20/50	20/40	20/30	20/25	20/20	20/200
**Axial length, mm**	26.81	21.81	16.21	22.91	24.04	27.55
**Preoperative status of the lens**	Phakia	Phakia	Pseudophakia	Phakia	Phakia	Phakia
**Surgical techniques employed**	Phacovitrectomy	Scleral Buckling	Vitrectomy	Phacovitrectomy	Phacovitrectomy	Phacovitrectomy
**Tamponade**	Air	None	Air	Air	Air	Air
**Baseline: posterior vitreous detachment**	+		+	+	+	

Overall, preoperative fundus examinations of the patients with new BRVO showed one of two patterns of retinal findings: the presence of a beaded blood vessel or the presence of a retinal tear along a large retinal vein.

In three eyes (cases 1, 2, and 3), a beaded or tortuous retinal vein was observed preoperatively. Multiple preoperative retinal dot hemorrhages were also observed around the abnormal retinal vein in two of the three cases. After surgical treatment of RRD, BRVO manifesting as multiple retinal hemorrhages in the areas distributed by these abnormal retinal veins was observed 1 week postoperatively in all three cases.

The other three eyes that developed new BRVO (cases 4, 5, and 6) all had a retinal tear along a large retinal vein on preoperative examination. During all three surgeries, strong adhesion was noted between the retinal vein, retinal tear, and vitreous, and a part of the retinal vein was resected to release the adhesion during surgery. There were no specific findings at postoperative week 1, but multiple retinal hemorrhages were observed at 4 weeks postoperatively in all three cases.

Across all six cases, the average time to onset was 15.2 days (range 0–29 days). The average aggravation duration was 85.3 days (range 67–115 days). The final visual acuity was logMAR 0.250 (Snellen 20/35, range 20/200 ~ 20/20). Details of these six cases are provided below.

### Case 1

**Fig. 1:**
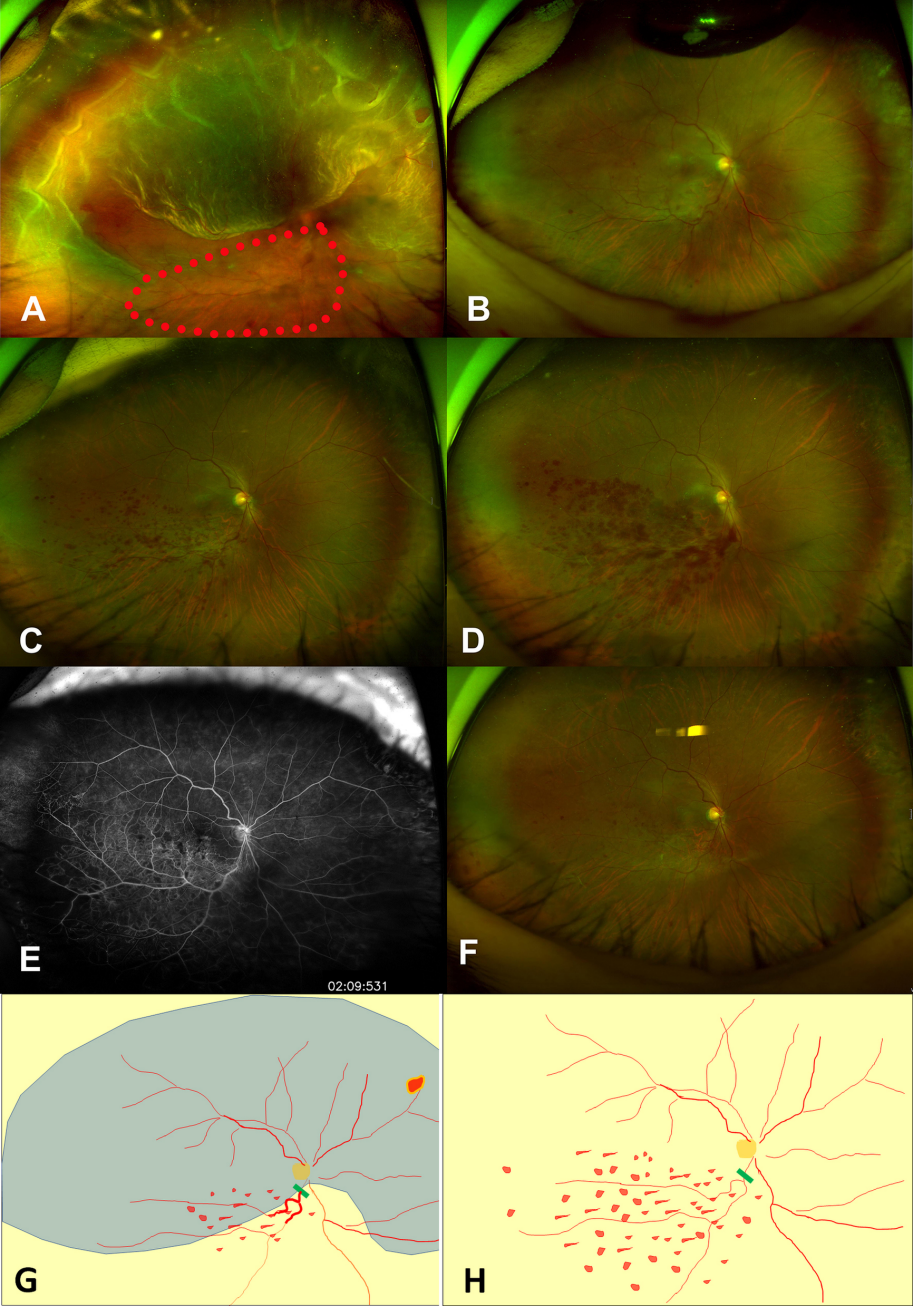
Series of fundus photographs of Case 1, a 48-year-old woman who presented with rhegmatogenous retinal detachment of the right eye invading from the 8 o’clock to 4 o’clock positions and involving the whole macula. Preoperatively, a beaded retinal vein with surrounding dot hemorrhage (red dots circle) was present inferior to the macula (**A**). A fundus photo taken at 10 days postoperatively showed tamponading air and the continued presence of the beaded retinal vein and adjacent dot hemorrhage around it (**B**). The dot hemorrhage appeared to have worsened in severity by postoperative week 4 (**C**) and to have worsened further at postoperative week 8 (**D**). Fluorescein angiography performed at 10 months postoperatively revealed no additional pathology (**E**). The dot hemorrhage was decreased in the fundus photo taken at 1 year postoperatively (**F**). A drawing of the preoperative fundus features (Fig. 1A) showed detached retina (blue color) and territory of the occluded retinal vein (**G**). Green bar indicates the presumed occluded lesion of retinal vein (**G**). A drawing of fundus features at 4 weeks after the surgery (Fig. 1D) (**H**)

A 48-year-old woman presented with RRD in the right eye invading from the 8 o’clock to the 4 o’clock positions and involving the whole macula. At presentation, her BCVA was 20/400 in the right eye. Untreated hypertension was noted (systolic pressure, 180 mmHg: diastolic pressure, 100 mmHg). Several dot hemorrhages and beaded blood vessels were observed in the inferior half of the retina (Fig. 1A). Vitrectomy with phacoemulsification was performed, followed by air tamponade. The multiple dot hemorrhages had worsened by postoperative day 10 (Fig. 1B) and continued worsening until postoperative day 70 (Figs. 1C, D), before improving and eventually resolving by 11 months postoperatively (Figs. 1E, F). A drawing (Figure G) explained that where retinal vein was blocked was within the detached retina.

### Case 2

**Fig. 2:**
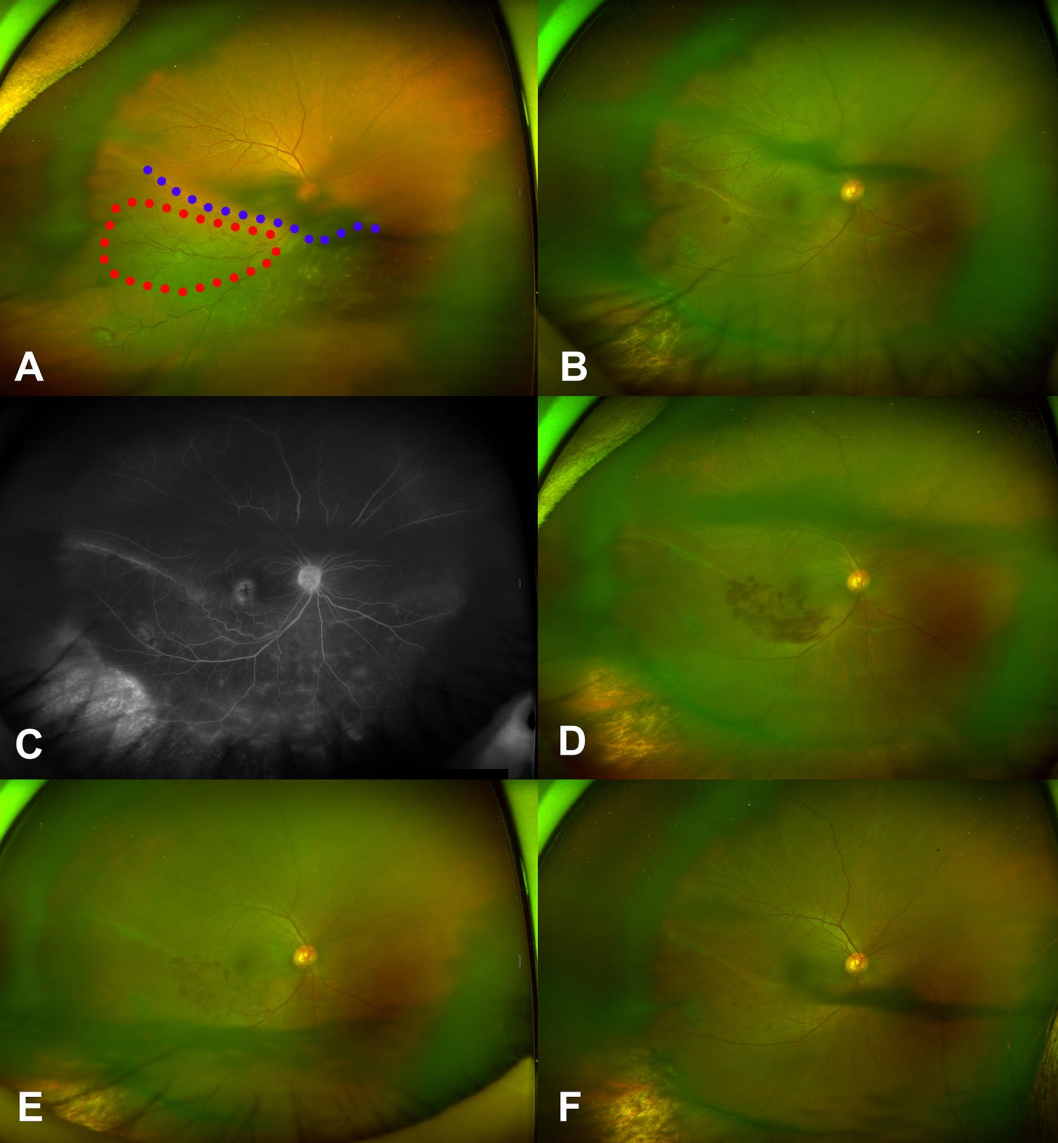
Series of fundus photos of Case 2 a 63-year-old woman who presented with retinal detachment of the right eye invading from the 2 o’clock to 8 o’clock positions of the retina and involving the fovea; a horseshoe tear was also noted at the 7 o’clock position preoperatively and the demarcation line (blue dots) crossed the macula obliquely from the 2 o’clock to 10 o’clock positions (**A**). The inferior retinal vein appeared tortuous and there was some evidence of dot hemorrhage (red dots circle) in the territory of the tortuous vein (**A**). Fundus photo taken at 8 weeks postoperatively showed that the detached retina had reattached after scleral buckling (**B**). The beaded retinal vein looked healthier than before the surgery, but the dot hemorrhage increased (**B**). Fluorescein angiography taken at 8 weeks postoperatively showed no pathology other than branch retinal vein occlusion and rhegmatogenous retinal detachment (**C**). The hemorrhage appears to have worsened at 15 weeks postoperatively (**D**). The hemorrhage appears to have improved in the fundus photo taken at 6 months postoperatively (**E**) and that taken at 1 year postoperatively (**F**)

A 63-year-old woman presented with decreased vision in her right eye, with BCVA in the right eye of 20/30. Fundus examination showed retinal detachment, invading from the 2 o’clock to the 8 o’clock positions and involving the fovea, and a horseshoe tear at the 7 o’clock position. Medical history was significant for medication treated dyslipidemia. The inferior retinal vein appeared tortuous, and there were some dot hemorrhages in the retina distributed by the tortuous vein (Fig. 2A). The patient underwent scleral buckling with drainage of the subretinal fluid.

Postoperatively, the beaded retinal vein appeared healthier than before the surgery, but the dot hemorrhages became more pronounced at 8 weeks postoperatively (Fig. 2B). FA performed at 9 weeks postoperatively showed no pathology other than BRVO and RRD (Fig. 2C). BRVO was diagnosed based on the distribution of multiple hemorrhages. The occlusion site of the retinal vein was presumed to be on the demarcation line of the RRD. The hemorrhages continued to worsen until postoperative week 15 (Fig. 2D). Subsequently, the flame-shaped hemorrhages decreased (Fig. 2E). Intravitreal bevacizumab was repeatedly injected (nine times) to control BRVO-induced macular edema. The hemorrhages had almost resolved at 14 months postoperatively (Fig. 2F).

### Case 3

**Fig. 3:**
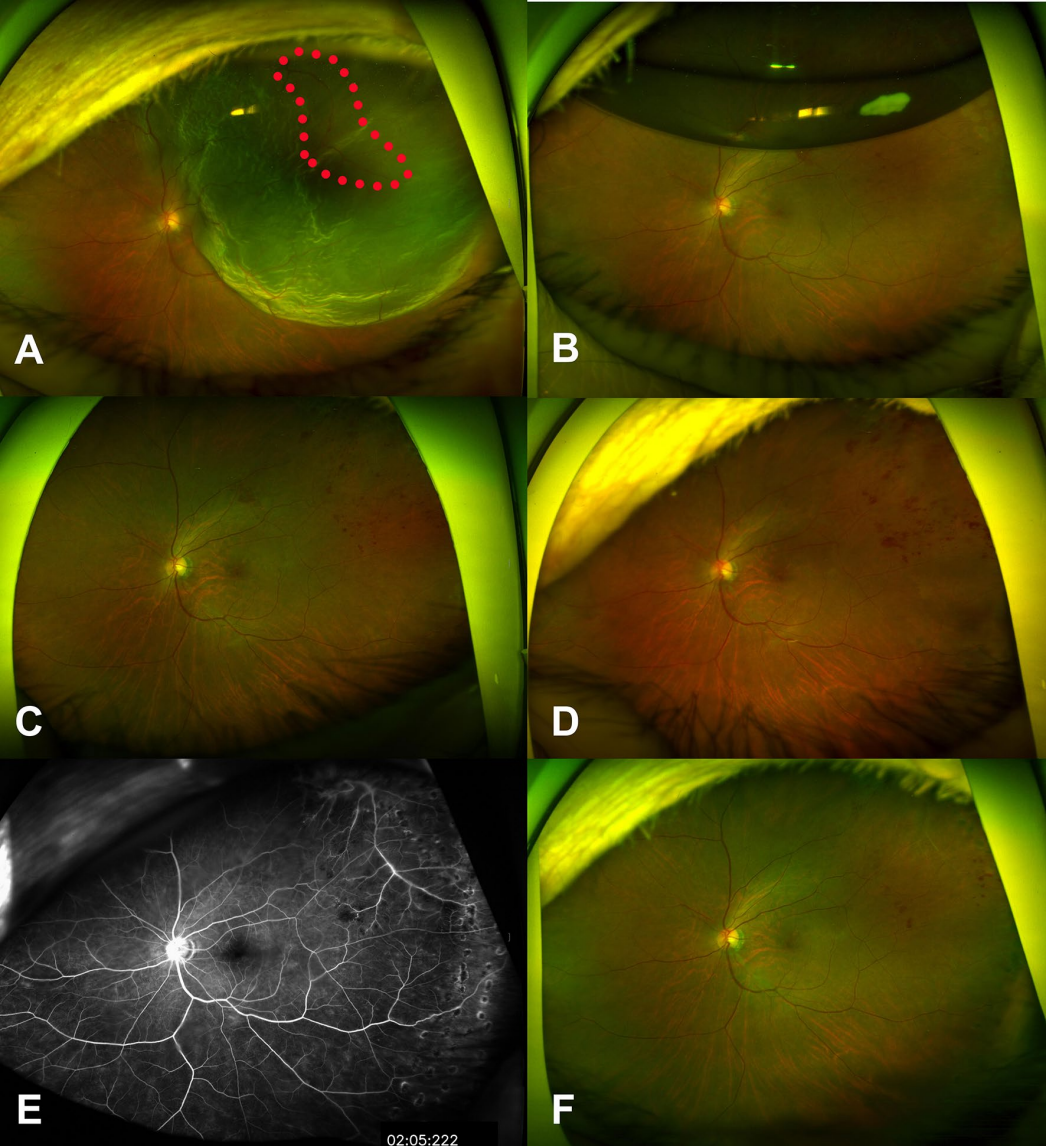
Series of fundus photos of Case 3, a 74-year-old woman who presented with rhegmatogenous retinal detachment of the left eye. Preoperative fundus photo showed bullous retinal detachment from the 11 o’clock to 4 o’clock positions involving the fovea and tortuous and beaded veins (red dots circle) were visible in the superotemporal quadrant (**A**). Fundus photo at 1 week postoperatively showed a retinal hemorrhage in the superotemporal quadrant, partially obscured by tamponading air (**B**). The hemorrhage increased in the fundus photo at 4 weeks postoperatively (**C**) and that taken at 9 weeks postoperatively (**D**). Fluorescein angiography at 9 weeks postoperatively revealed no additional pathology (**E**). The hemorrhage appeared to have improved in the fundus photo taken at 1 year postoperatively (**F**)

A 74-year-old woman presented with RRD in her left eye, with a BCVA of 20/4000 in the left eye. A fundus examination showed bullous retinal detachment from the 11 o’clock to 4 o’clock positions involving the fovea with a tear at the 1 ~ 2 o’clock position. Tortuous and beaded retinal veins were visible preoperatively in the superotemporal quadrant (Fig. 3A). Medical history was significant for hypertension that was well controlled with medication. Phacovitrectomy was performed, followed by air tamponade. Multiple dot hemorrhages were observed 7 days after vitrectomy (Fig. 3B) and were worsened at 2 months postoperatively (Figs. 3C, D). The hemorrhages were in the territory of the preoperative tortuous and beaded retinal veins. There was no specific pathology other than RRD or BRVO on fluorescein angiography (Fig. 3E). One year after surgery, the hemorrhages had nearly resolved (Fig. 3F).

### Case 4

**Fig. 4:**
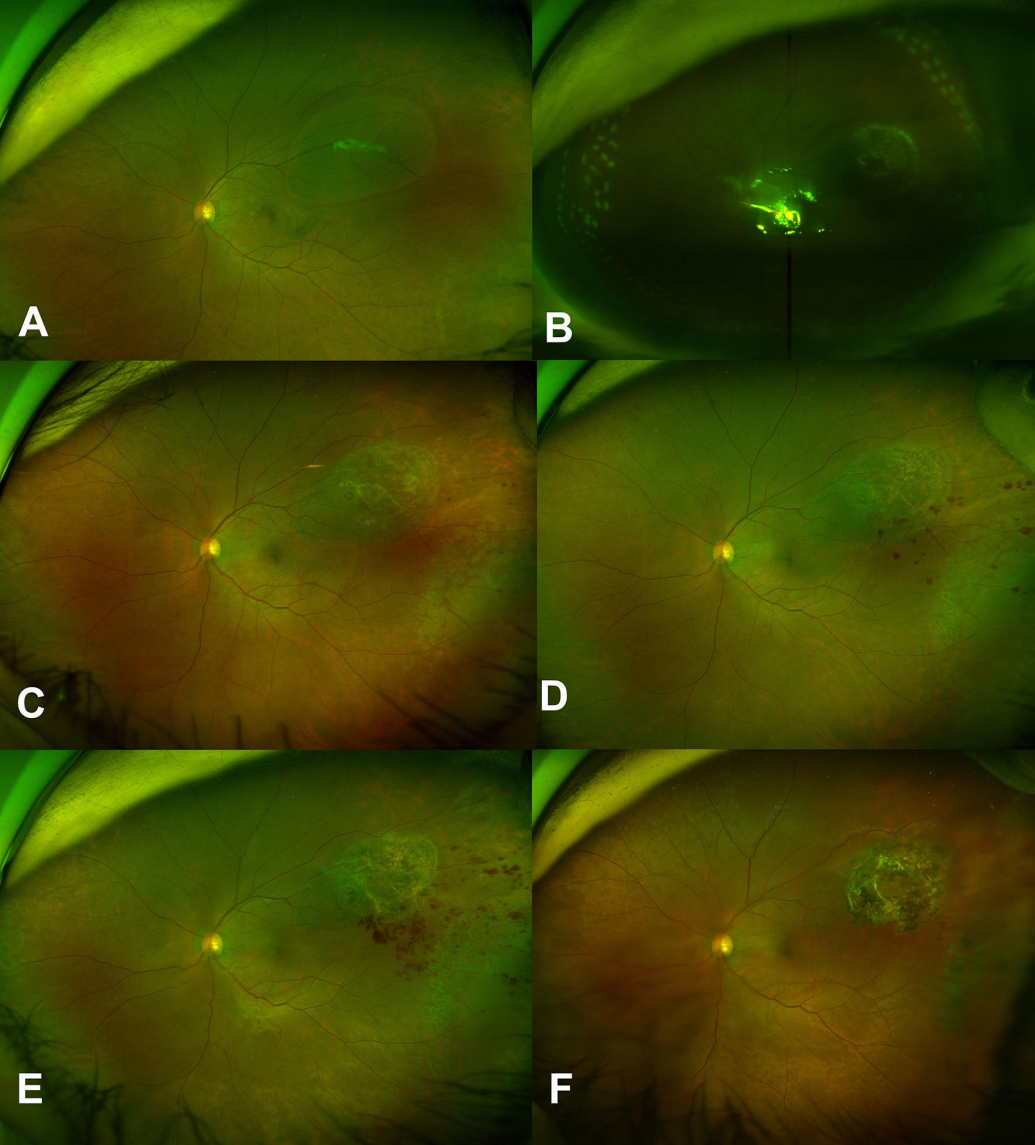
Series of fundus photos of Case 4, a 63-year-old woman who presented with rhegmatogenous retinal detachment of the left eye. A preoperative fundus photo showed localized retinal detachment at the mid-peripheral retina of the superotemporal quadrant and a tear was positioned along the large superior temporal retinal vein and appeared strongly attached to the vitreous (**A**). The retinectomy site, surrounding fresh laser burns, and tamponading air were visible in the fundus photo on postoperative day one (**B**). A retinal dot hemorrhage was visible in the temporal periphery at four weeks postoperatively (**C**) and had progressively worsened at eight (**D**) and ten (**E**) weeks postoperatively. The retinal dot hemorrhage was almost completely resolved at one year follow up (**F**)

A 63-year-old woman presented with RRD invading the mid-peripheral retina of the superotemporal quadrant of her left eye. The BCVA of the left eye was 20/25. A tear was located on the large superior retinal vein, there seemed strong adhesion between the tear and the vein. No specific preoperative abnormalities such as tortuous vein or dot hemorrhage were visible (Fig. 4A). The patient underwent an uncomplicated vitrectomy with phacoemulsification. The retina and retinal vein around the tear were removed to release the adhesion among them. Air tamponade was subsequently performed. A rounded retinectomy was noticed among the fresh laser burns the next day (Fig. 4B). Several dot hemorrhages were observed in the temporal and peripheral areas at four weeks postoperatively (Fig. 4C). The multiple dot hemorrhages worsened through postoperative week 10 (Figs. 4D, E) before improving and almost resolving by 18 months postoperatively (Fig. 4F).

### Case 5

**Fig. 5:**
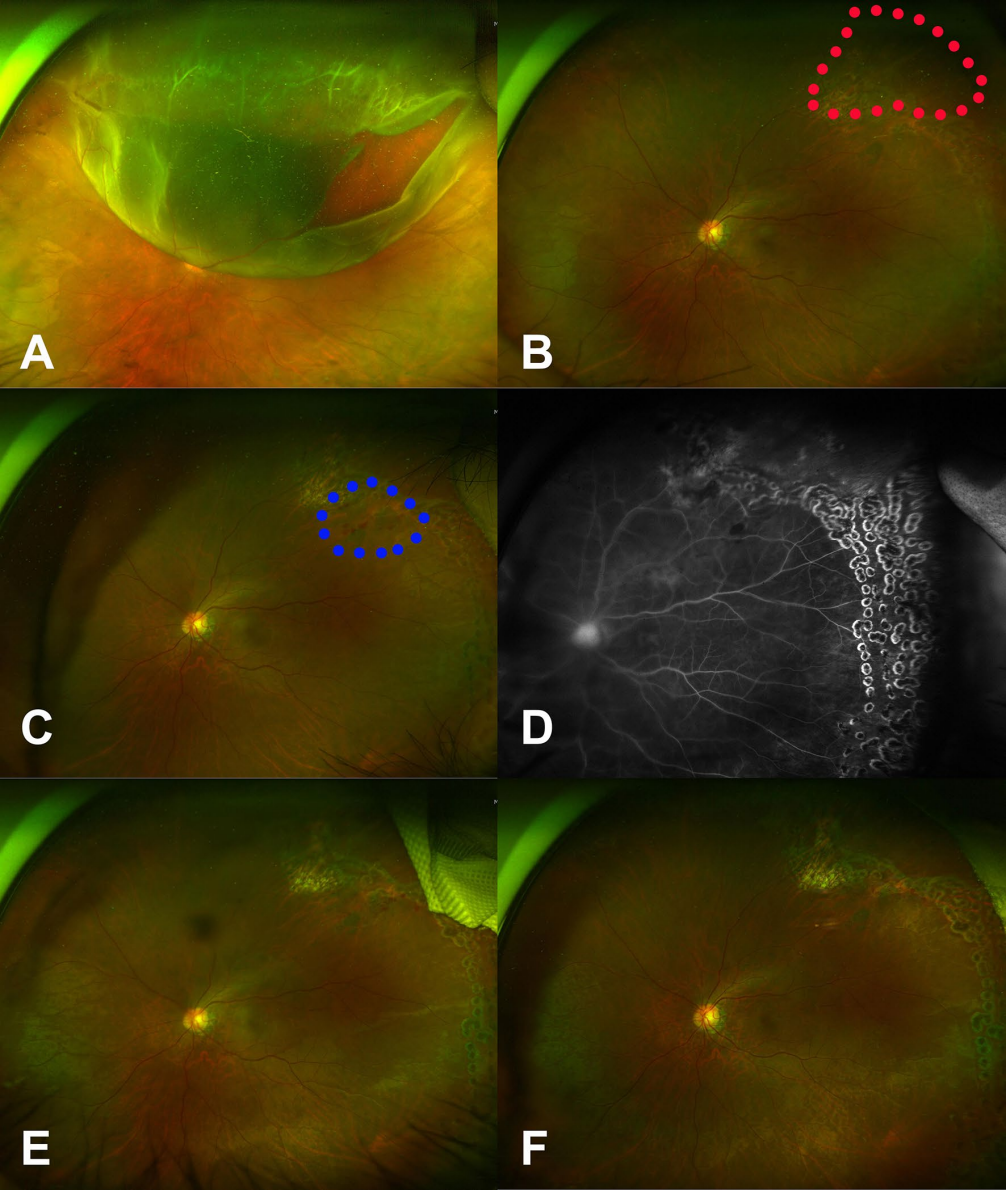
Series of fundus photos of Case 5, a 64-year-old woman who presented with rhegmatogenous retinal detachment of the left eye. A preoperative fundus photo showed retinal detachment invading the retina from the 10 o’clock to 2 o’clock positions and a retinal tear along the superior temporal large retinal vein and the retinal tear appeared strongly attached to the vitreous and retinal vein (**A**). Red dots circle indicated the retinectomy lesion. No distinct retinal hemorrhage or abnormality of the retinal circulation was noted at 2 weeks postoperatively (**B**). Multiple retinal hemorrhages (Blue dots circle) were first noted around the retinectomy site 4 weeks postoperatively (**C**). Some diffuse leakage, including leakage of the optic disc, was noted on fluorescein angiography at 12 weeks postoperatively (**D**). The retinal hemorrhage appeared worsened in the fundus photo taken at 18 weeks postoperatively (**E**). The hemorrhage had almost resolved at 1 year postoperatively (**F**)

A 64-year-old woman presented with decreased vision in her right eye, with a BCVA of 20/30 in the right eye. RRD was noted invading the retina from the 10 o’clock to 2 o’clock positions. The retina was torn along the large superior retinal vein. No specific abnormalities such as tortuous veins or dot hemorrhages were noted (Fig. 5A). She had no history of hypertension or dyslipidemia. A phacovitrectomy was performed. The retina and retinal vein around the tear were removed to release adhesions among them and the vitreous cavity was filled with air. No specific abnormality suspicious for BRVO was noted at postoperative week 2 (Fig. 5B). At 1 month postoperatively, multiple dot hemorrhages were noted near the resected vein (Fig. 5C) and there was no specific pathology other than RRD or BRVO on fluorescein angiography (Fig. 5D). The hemorrhage subsequently improved (Fig. 5E) and resolved by 18 months postoperatively (Fig. 5F).

### Case 6

**Fig. 6:**
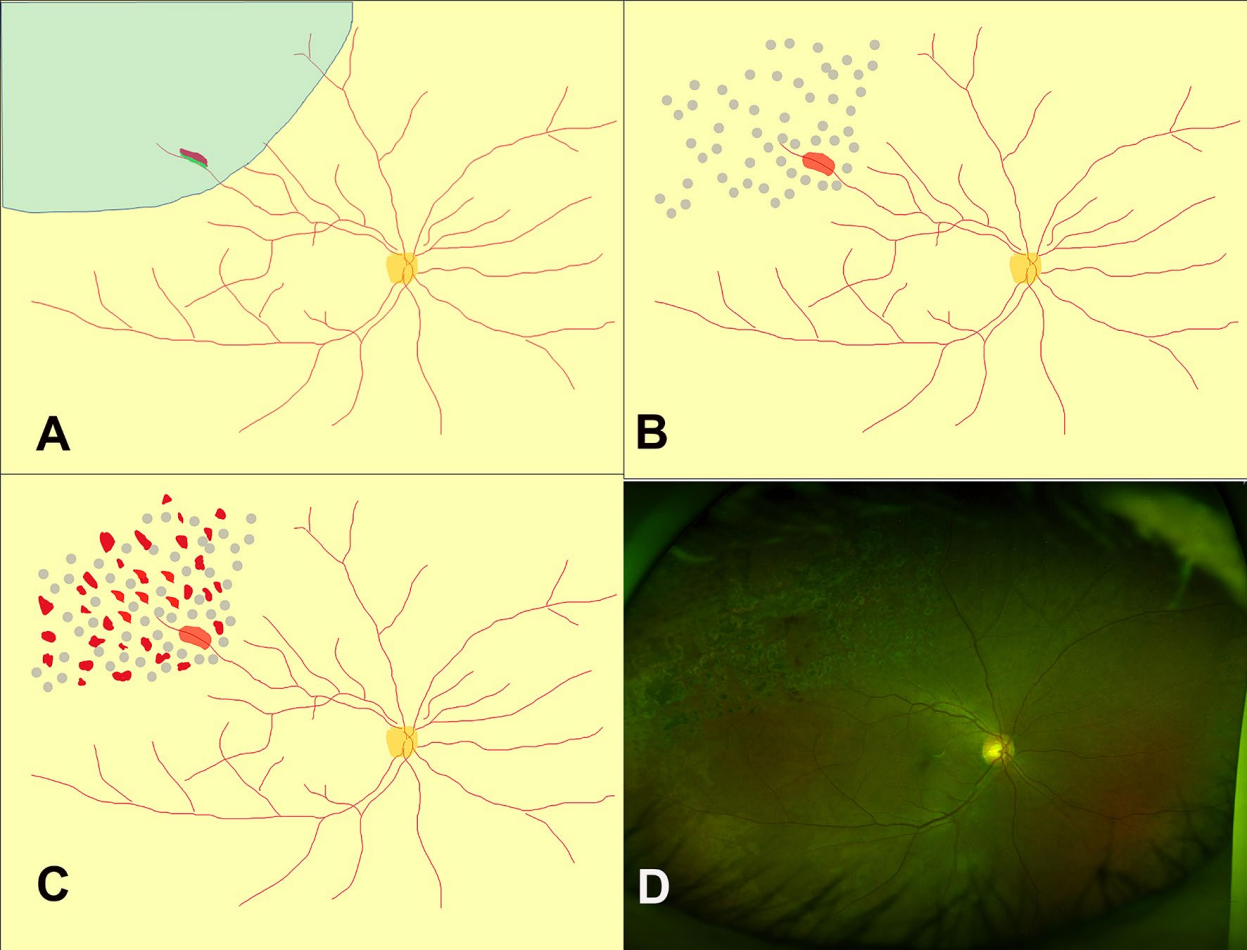
Retinal findings of Case 6, a 48-year-old man who presented with rhegmatogenous retinal detachment of the right eye. A drawing of the preoperative fundus features illustrates the observed retinal detachment invading retina from the 10 o’clock to 1 o’clock positions and the retinal tear along the retinal vein in the superotemporal region (**A**). No retinal hemorrhages were visible at 1 week postoperatively (**B**). A retinal hemorrhage was present in the superotemporal region at 4 weeks postoperatively (**C**). The retinal hemorrhage had almost resolved at 18 months postoperatively (**D**)

A 48-year-old man presented with decreased vision in his left eye, with a BCVA of 20/200 in the left eye. He had no history of hypertension or dyslipidemia. Retinal detachment was observed from the 10 o’clock to 1 o’clock positions with a large, 4-disc diameters (DD), tear along the superotemporal retinal vein (Fig. 6A). The patient underwent vitrectomy with phacoemulsification. The retina and retinal vein around the tear were removed to release the adhesions among them (Fig. 6B) and air tamponade was subsequently performed. At 1 month postoperatively, a retinal hemorrhage was observed around the tear (Fig. 6C). The hemorrhage improved and resolved by 18 months postoperatively (Fig. 6D).

## Discussion

The occurrence of BRVO in the six cases reported here was presumed to be likely related to RRD itself or to surgical treatment of RRD rather than coincidental. Given that incidence of RVO is reportedly 48.31–74.16 per 100,000 person-years in Korea [14–17], the incidence of RVO in this cohort was about 10 times higher. On top of that, all six BRVO occurred within 4 weeks after surgery.

Detailed below are two different presumed mechanisms that may explain the elevated incidence of BRVO after RRD surgery.

### Mechanism I (Cases 1, 2, and 3)

In three of the eyes, all belonging to patients with hypertension or dyslipidemia, a beaded retinal vein was seen preoperatively and a flame-shaped hemorrhage in the territory of the beaded retinal vein was seen within 7 days postoperatively in these cases, the beaded retinal vein was likely already partially occluded prior surgery, and the occurrence of BRVO was related to RRD itself rather than the surgical procedure.

**Fig. 7:**
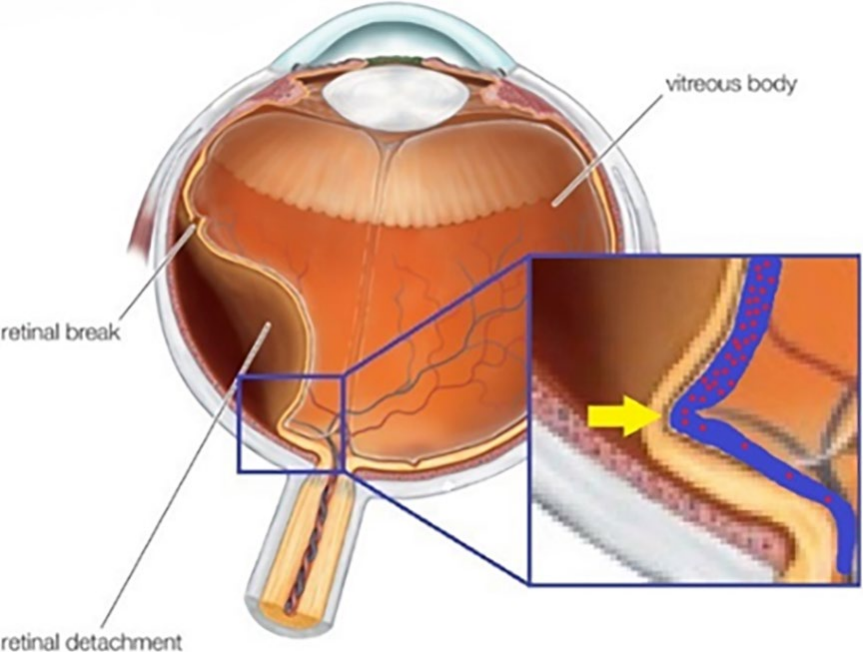
Proposed mechanism I of retinal vein occlusion after rhegmatogenous retinal detachment (RRD) (cases 1, 2, and 3). Kinking of the retina is common in RRD and causes folding of the retinal vein. Folding of the retinal vein may increase the likelihood of retinal vein stasis, especially in patients with older age, hypertension, or dyslipidemia

Virchow’s triad describes three factors that contribute to the development of venous thrombosis: hypercoagulability, stasis and endothelial injury [18]. It is presumed that the retinal veins were damaged like Virchow’s triad: hypercoagulability related to pre-existing atherosclerosis, venous stasis by kinking of the retinal vein, or endothelial injury by ischemia related to detached retina [19]. Elements of the case presentations support each of these three pathways. First, a kink of the retinal vein accompanied by a kink in the retina might induce venous stasis. (Fig. 7). In case 2, the site of occlusion was on the demarcation line of the retinal detachment, which had been formed by a long-standing kink of the retina. Second, cases 1, 2, and 3 involved hypertension or hyperlipidemia, and relatively advanced age (mean age 61.7 years) which are all well-known risk factors of atherosclerosis. Third, blood vessels are vulnerable to hypoxic conditions and the significant decrease in blood flow in RRD may cause endothelial cell ischemia [19].

### Mechanism II (cases 4, 5, and 6)

Three eyes with vitrectomy were included in this category. Unlike Cases 1, 2, and 3, no beaded retinal veins were noted preoperatively, nor did the patients have hypertension or dyslipidemia. The flame-shaped hemorrhages occurred somewhat later, around 4 weeks postoperatively.

Each tear was located on a large retinal vein. The tears occurred more posteriorly than the typical RRD. The direction of the tear was along the direction of the vein. In all three cases, strong adhesions requiring surgical resection were observed between the retinal vein, retina, and vitreous.

**Fig. 8:**
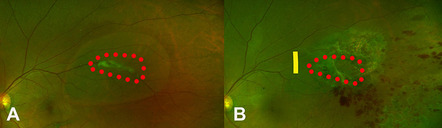
Proposed mechanism II of retinal vein occlusion after rhegmatogenous retinal detachment (RRD) (case 4, 5, and 6). Fundal photo (Case 4) showed that the vitreous was strongly adherent to a large retinal vein where retinal tears (red dots circle) occurred (**A**) New branch retinal vein occlusion occurred near this site after surgery. The presumed obstructive lesion (yellow line) was just proximal to the retinectomy site (red dots circle) (**B**)

Direct injury from intraoperative resection is presumed to have caused venous occlusion. Interestingly, the occlusive point was not coincident with the dissected vein; rather, it was about 0.5 DD proximal to that point. Vessel wall defects presumably could affect the function of the proximal part (Fig. 8).

To the best of our knowledge, only one case report to date has described BRVO occurring after RRD surgery [10]. The patient was a healthy 26-year-old man without any cardiovascular problems. BRVO occurred 5 days after vitrectomy combined with scleral buckling for RRD. No mechanistic explanations for BRVO occurring after RRD surgery were explored in that single report.

### Limitations

This cohort was relatively small and derived from a single center. Postoperative ultra-wide fundus photographs were reviewed for all patients, but preoperative ultra-wide fundus photographs were not available for all patients. Therefore, the odds ratio of these preoperative findings (ischemic retina or abnormal vitreovenous adhesion) were not analyzed.

## Conclusion

The postoperative period following RRD surgery was associated with an elevated incidence of BRVO, which suggest RRD itself or RRD surgery may be risk factors for BRVO. Two possible mechanisms for this association include kinks of the retinal vein on the detached retina and retinal vein damage incurred during vitrectomy. Although occlusion of the retinal vein seemed to have occurred before or during the surgery, flame shape hemorrhages became distinct several weeks later and then worsened for a while.

## Data Availability

All data generated or analyzed during this study are included in this published article.
